# Targeted disruption of the K-*Ras* oncogene in an invasive colon cancer cell line down-regulates urokinase receptor expression and plasminogen-dependent proteolysis

**DOI:** 10.1038/sj.bjc.6690616

**Published:** 1999-08

**Authors:** H Allgayer, H Wang, S Shirasawa, T Sasazuki, D Boyd

**Affiliations:** Departments of Cancer Biology, The University of Texas MD Anderson Cancer Center, 1515 Holcombe Blvd, PO Box 108, Houston, TX 770 30, USA; Department of Genetics, Kyushu University, Fukuoka, Japan; Department of Surgery, Klinikum Grosshadern, Ludwig-Maximillians University, Munich, Germany

**Keywords:** u-PAR, K-Ras, colon cancer, knockout, proteolysis

## Abstract

The urokinase receptor, overexpressed in invasive colon cancer, promotes tumour cell invasion. Since K-*Ras* is activated in many colon cancers, we determined if urokinase receptor overexpression is a consequence of this activated oncogene. Accordingly, urokinase receptor expression was compared in HCT 116 colon cancer cells containing either a mutation-activated K-*Ras* or disrupted for this oncogene (by homologous recombination). HCT 116 cells containing the disrupted K-*Ras* oncogene expressed between 50 and 85% less urokinase receptor protein compared with the parental HCT 116 cells. Reduced urokinase receptor expression in cells containing the disrupted mutated K-*Ras* was not due to a physical impairment of the urokinase receptor gene since phorbol ester treatment was inductive for its expression. Constitutive urokinase receptor expression in HCT 116 cells required an intact AP-1 motif in the promoter (at –184) and electrophoretic mobility shifting assays indicated less c-*Jun*, *Jun*D, c-*Fos* and *Fra*-1 bound to this motif in the K-*Ras*-disrupted cells. Since the urokinase receptor accelerates proteolysis, laminin degradation was compared in cells containing the mutation-activated and disrupted K-*Ras* oncogene. The latter cells displaying fewer urokinase receptors, degraded 80% less laminin. This is the first study to demonstrate a role for K-*Ras* as a regulator of the constitutive expression of the urokinase receptor. © 1999 Cancer Research Campaign


*British Journal of Cancer* (1999) **80**(12), 1884–1891
© 1999 Cancer Research Campaign
Article no. bjoc.1999.0616

### Targeted disruption of the K-*Ras* oncogene in an invasive colon cancer cell line down-regulates urokinase receptor expression and plasminogen-dependent proteolysis

**H Allgayer[1,3], H Wang[1], S Shirasawa[2], T Sasazuki[2] and D Boyd[1]**

[1]Departments of Cancer Biology, PO Box 108, The University of Texas MD Anderson Cancer Center, 1515 Holcombe Blvd, Houston, TX 770 30, USA; [2]Department of Genetics, Kyushu University, Fukuoka, Japan; [3]Present address: Department of Surgery, Klinikum Grosshadern, Ludwig-Maximillians University, Munich, Germany

**Summary** The urokinase receptor, overexpressed in invasive colon cancer, promotes tumour cell invasion. Since K-*Ras* is activated in many colon cancers, we determined if urokinase receptor overexpression is a consequence of this activated oncogene. Accordingly, urokinase receptor expression was compared in HCT 116 colon cancer cells containing either a mutation-activated K-*Ras* or disrupted for this oncogene (by homologous recombination). HCT 116 cells containing the disrupted K-*Ras* oncogene expressed between 50 and 85% less urokinase receptor protein compared with the parental HCT 116 cells. Reduced urokinase receptor expression in cells containing the disrupted mutated K-*Ras* was not due to a physical impairment of the urokinase receptor gene since phorbol ester treatment was inductive for its expression. Constitutive urokinase receptor expression in HCT 116 cells required an intact AP-1 motif in the promoter (at –184) and electrophoretic mobility shifting assays indicated less c-*Jun*, *Jun*D, c-*Fos* and *Fra*-1 bound to this motif in the K-*Ras*-disrupted cells. Since the urokinase receptor accelerates proteolysis, laminin degradation was compared in cells containing the mutation-activated and disrupted K-*Ras* oncogene. The latter cells displaying fewer urokinase receptors, degraded 80% less laminin. This is the first study to demonstrate a role for K-*Ras* as a regulator of the constitutive expression of the urokinase receptor.

**Keywords**: u-PAR; K-Ras; colon cancer; knockout; proteolysis

The urokinase-type plasminogen activator (u-PA) is a serine protease that converts the inert zymogen plasminogen into plasmin ,a protease with broad substrate specificity (Robbins et al, 1967; Nielsen et al, 1982). Urokinase can bind specifically and with high affinity ($K_D \simeq 0.5$ nM) to a 45–60 kDa, heavily glycosylated, cell surface receptor (u-PAR) (Vassalli et al, 1985; Stoppelli et al, 1986) comprised of three similar repeats of approximately 90 residues each (Behrendt et al, 1991; Riittinen et al, 1996). The aminoterminal domain binds the plasminogen activator with the carboxyterminus domain anchoring the binding protein to the cell surface via a glycosyl–phosphatidylinositol chain (Behrendt et al, 1991; Riittinen et al, 1996). The 7 exon u-PAR gene is located on chromosome 19q13 (Vagnarelli et al, 1992; Casey et al, 1994) and transcription of the gene yields a 1.4-kb mRNA or an alternatively spliced variant lacking the membrane attachment peptide sequence (Roldan et al, 1990; Pyke et al, 1993). The amounts of u-PAR are controlled mainly at the transcriptional level through 398 base pairs of upstream sequence, but altered message stability and receptor recycling may represent other means of controlling the amount of this gene product at the cell surface (Lund et al, 1995; Lengyel et al, 1996; Shetty et al, 1997; Nykjaer et al, 1997).

*Received 17 November 1998*
*Revised 4 March 1999*
*Accepted 9 March 1999*

*Correspondence to:* D Boyd

The u-PAR is a multi-functional molecule. First, urokinase bound to the u-PAR activates plasminogen at a much faster rate than fluid-phase plasminogen activator (Ellis et al, 1991; Higazi et al, 1995). Second, the binding site clears urokinase–inhibitor complexes from the extracellular space (Cubellis et al, 1990; Conese et al, 1994) through a mechanism involving the α2 macroglobulin receptor. Third, it is now evident that the u-PAR interacts with the extracellular domain of integrins to connect to the cytoskeleton thereby mediating cell adhesion and migration (Bohuslav et al, 1995; Wei et al, 1996; Yebra et al, 1996).

In cancer, the u-PAR plays a prominent role in tumour cell invasion and metastasis. Earlier studies have shown that the overexpression of a human u-PAR cDNA increased the ability of human osteosarcoma cells to invade into an extracellular matrix-coated porous filter (Kariko et al, 1993). Conversely, down-regulating u-PAR levels using either antisense expression constructs, oligonucleotides, or synthetic compounds reduced the ability of Hep3 squamous cell carcinoma, breast cancer cells and transformed fibroblasts to demonstrate an invasive phenotype in vitro and in vivo (Kook et al, 1994; Quattrone et al, 1995). Similarly, Wilhelm et al (1994) demonstrated that soluble u-PAR used as a scavenger inhibited the in vitro invasion of ovarian cancer cells. In clinical studies, u-PAR-positive tumour cells in the bone marrow of gastric cancer patients is an indicator of a metastatically-relevant population in a pool of minimal residual tumour cells (Allgayer et al, 1997).

We demonstrated previously that cultured colon cancer cell lines displaying a large number of u-PAR at the cell surface (>$10^5$ per cell) were more invasive in vitro compared with other colon cancer cell lines equipped with tenfold fewer binding sites (Hollas et al, 1991) and that interfering with the binding of urokinase with its receptor to the former group reduced their ability to degrade laminin (Schlechte et al, 1989; Hollas et al, 1991) and invade in vitro. These data were consistent with in situ hybridization studies localizing u-PAR mRNA to cancer cells in invasive foci of colon adenocarcinomas (Pyke et al, 1991). As a clinical corollary, Ganesh and co-workers (Ganesh et al, 1994) showed that in colon cancer patients, a high u-PAR protein level is a predictor of a poor 5-year outcome. How then is the u-PAR gene overexpressed in colon cancer? It is widely documented that K-*Ras* is mutated at a high (50%) rate in colon cancer (Ahnen et al, 1998). Considering this observation, we undertook a study to determine if the high u-PAR protein in a cultured colon cancer cell line (HCT 116) characterized as having a mutation-activated K-*Ras* ($Gly^{13}$ to $Asp^{13}$) (Shirasawa et al, 1993) was due to this activated oncogene. Towards this end, we compared u-PAR expression in HCT 116 cells containing the mutation-activated K-*Ras* with HCT 116 cells in which this gene had been disrupted by homologous recombination (Shirasawa et al, 1993). We report that u-PAR expression, as measured at the protein/mRNA levels as well as by laminin degradation, is decreased in HCT 116 cells in which the K-*Ras* oncogene has been 'knocked out' and that this is probably largely a consequence of decreased binding of c-*Jun* and c-*Fos* to a regulatory AP-1-binding motif located in the promoter region of the u-PAR gene.

### MATERIALS AND METHODS

### Cell lines

HCT 116 cells and the mutation K-*Ras*-knocked out cells (HKh-2, HKe-3 and HK2-8) were grown in McCoys 5A medium supplemented with, or without, 10% fetal bovine serum (FBS). The disruption of the mutation-activated K-*Ras* in HCT 116 cells thereby generating HKh-2, Hke-3 and HK2-8 clones was as described previously (Shirasawa et al, 1993).

### Vectors and antibodies

The u-PAR CAT/luciferase reporters consisted of 449 base pairs of sequence (Wang et al, 1995) stretching from –398 to +51 (relative to the transcription start site) cloned into pCAT-Basic vector or pGL3 (Promega, Madison, WI, USA). The mutated AP-1 u-PAR CAT reporter (AP-1 distal mt u-PAR CAT) has been described previously (Lengyel et al, 1996). Oligonucleotides were purchased from Genosys Biotechnologies (The Woodlands, TX, USA). Supershifting antibodies were obtained from Santa Cruz Biotechnology (Santa Cruz, CA, USA). The activated c-Ha-*ras* expression construct included a 6.6-kb *Bam*HI fragment from the activated c-Ha-$ras^{EJ}$ oncogene from T24 bladder carcinoma cells cloned in a pSV2neo plasmid (Nicolson et al, 1990).

### Preparation of nuclear extracts and EMSA

Nuclear extracts and electrophoretic mobility shift assay (EMSA) were carried out as described elsewhere (Lengyel et al, 1996). The oligonucleotide used corresponding to the sequence of the u-PAR promoter spanning nucleotides –199/–170. EMSA was performed using nuclear extract (15 μg), 0.6 μg of poly dI/dC and ($2 \times 10^4$ cpm) of a T4 polynucleotide kinase-labelled ($\gamma^{32}$P) ATP oligonucleotide.

### Reporter assays

Cells were transfected at 60% confluency using poly-L-ornithine as described previously (Nead and McCance, 1995). Where indicated, transient transfections were performed in the presence of a luciferase expression vector (4 μg) and transfection efficiencies determined by assaying for luciferase activity. CAT activity was measured as described previously (Lengyel et al, 1996). The amount of acetylated [$^{14}$C]chloramphenicol was determined using a Storm 840 Phosphorimager (Molecular Dynamics, Sunnyvale, CA, USA) using ImageQuant software. Reporter assays using the u-PAR promoter fused to a luciferase reporter were as described by us previously (Allgayer et al, 1999).

### Western blotting and ELISA for u-PAR protein

Cells were extracted into a buffer (10 mM Tris pH 7.4, 0.15 M sodium chloride, 1% Triton X-100, 0.5% NP-40, 20 μg $ml^{-1}$ aprotinin, 1 mM phenylmelthylsulphonyl fluoride, 1 mM EGTA, 1 mM EDTA) for 10 min at 4°C. Insoluble material was removed by centrifugation and 750 μg protein of cell extract immunoprecipitated at 4°C for 16 h with 0.25 μg of a polyclonal anti-u-PAR antibody and protein A agarose beads. The polyclonal antibody (kindly provided by Dr Andrew Mazar, Angstrom Pharmaceuticals, San Diego, CA, USA) was raised in rabbits against amino acids 1–281 of the human u-PAR and purified on a Sepharose-immobilized u-PAR column. The immunoprecipitated material was subjected to standard Western blotting (Burnette, 1981) and the blot probed with 5 μg $ml^{-1}$ of an anti-u-PAR monoclonal antibody (#3931 American Diagnostica, Greenwich, CT, USA) and an HRP-conjugated goat anti-mouse IgG. Bands were visualized by enhanced chemiluminescence (Amersham, Arlington Heights, IL, USA).

u-PAR protein determinations by enzyme-linked immunosorbent assay (ELISA) were performed as described by the manufacturer (American Diagnostica, Greenwich, CT, USA).

### Northern blotting

The level of steady state urokinase receptor transcript was determined by Northern analysis (Lengyel et al, 1996). Total cellular RNA was extracted from 90% confluent cultures using 5.0 M guanidinium isothiocyanate and purified on a caesium chloride cushion (5.7 M) by centrifugation at 150 000 ***g*** for 20 h. Purified RNA was electrophoresed in a 1.5% agarose-formaldehyde gel and transferred to Nytran-modified nylon by capillary action using 10 × sodium–saline citrate (SSC). The Northern blot was probed at 42°C with a random primed radiolabelled 0.65-kb cDNA specific for u-PAR mRNA (Roldan et al, 1990). The blots were washed at 65°C using 0.25 × SSC (SSC = 0.15 M sodium chloride, 15 mM sodium citrate, pH 7.4) in the presence of 0.75% sodium dodecyl sulphate (SDS). Loading efficiencies were checked by reprobing the blot with a radioactive cDNA which hybridizes with the glyceraldehyde-3-phosphate dehydrogenase (GAPDH) mRNA.

© 1999 Cancer Research Campaign

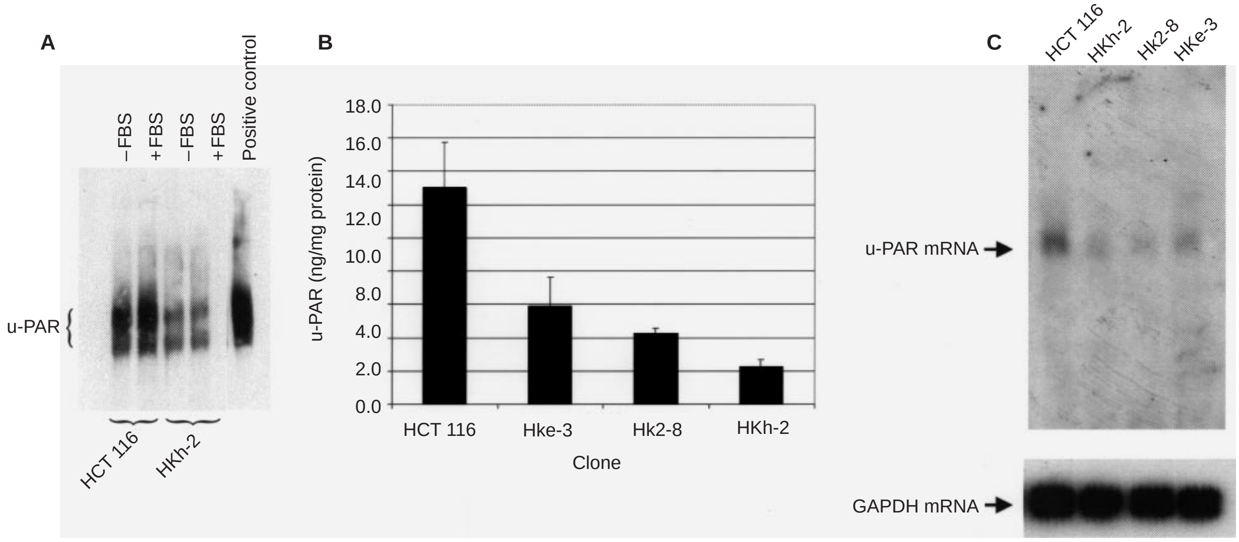

**Figure 1** Reduced expression of u-PAR in HCT 116 cells containing a disrupted K-Ras. (**A**) Cells were grown to 95% confluence in McCoys 5A medium supplemented with (+ FBS) or without (–FBS) 10% FBS. Subsequently, cells were extracted, equal protein amounts immunoprecipitated with a polyclonal anti-u-PAR antibody and the material subjected to Western blotting using a monoclonal anti-u-PAR antibody. Immunoreactive bands were visualized by enhanced chemiluminescence. The positive control consists of cellular extract from the RKO cell line which displays 300 000 binding sites per cell (Boyd et al, 1988). (**B**) Cells were grown to 95% confluence in 10% FBS, extracted and assayed for u-PAR protein by an ELISA. (**C**) Cells were grown as described for (**B**), RNA extracted and purified. Purified RNA was analysed by Northern blotting using a 0.65 kb cDNA corresponding to the u-PAR mRNA. The blot was reprobed with a GAPDH cDNA. The data are typical of duplicate experiments

### Laminin degradation assays

These were carried out as described previously (Schlechte et al, 1989). Cells were harvested with 3 mM EDTA–PBS (phosphate-buffered saline), washed twice and seeded (100 000 cells) on radioactive laminin-coated (2 µg per dish) dishes. The cells were allowed to attach overnight. Subsequently, cell surface urokinase receptors were saturated with 5 nM urokinase and the cells washed extensively to remove the unbound protease. The cells were then replenished with serum-free medium with, or without, 10 µg $ml^{-1}$ plasminogen (final concentration). After varying times at 37°C, aliquots of the culture medium were withdrawn and counted for radioactivity. Solubilized laminin represents the degraded glycoprotein (Schlechte et al, 1989).

### RESULTS

### Effect of a disrupted activated K-*Ras* on u-PAR mRNA/protein

HCT 116 colon cancer cells contain an activated K-*Ras* (Buard et al, 1996). To determine the role of this activated oncogene in regulating u-PAR expression, the amount of the u-PAR protein/mRNA was compared in parental HCT 116 cells and their counterparts (HKh-2, Hke-3 and HK2-8) in which the activated K-*Ras* was disrupted by homologous recombination (Shirasawa et al, 1993). In Western blotting, reactive bands ($M_r$ 45–60 kDa) indistinguishable in size to the u-PAR receptor were detected using cellular extracts from HCT 116 cells (Figure 1A). The diffuse nature of the bands probably reflects the glycosylation state of the u-PAR protein (Moller et al, 1993). Cellular extracts generated with the clone HKh-2, in which the mutation-activated K-*Ras* had been 'knocked out', indicated substantially less of the u-PAR protein when compared with the parental HCT 116 cells. This difference was independent of whether the cells were propagated in serum-free medium (–FBS) or McCoys medium supplemented with 10% FBS (+ FBS). Extracts from HCT 116 and three independently derived clones in which the activated K-*Ras* had been 'knocked out' were also analysed by a commercially available ELISA for u-PAR protein (Figure 1B). Whereas HCT 116 cells contained 13.0 ± 2.7 ng u-PAR $protein^{-1}$ $mg^{-1}$ protein, this value was reduced by over 50% for clone Hke-3 (5.9 ± 1.5 ng $mg^{-1}$) and up to 80% for the HKh-2 clone (2.3 ± 0.4 ng $mg^{-1}$). It is presently unclear as to why u-PAR expression was not uniformly reduced in the three clones. This is unlikely to be due to different levels of expression of the activated K-*Ras* since all clones were verified as being disrupted at this allele as demonstrated by site-specific hybridization analysis, Southern blotting and reverse transcription polymerase chain reaction (RT-PCR) (Shirasawa et al, 1993).

To corroborate the u-PAR protein data, RNA was extracted and purified from the cells and analysed for steady-state u-PAR mRNA by Northern blotting (Figure 1C). The three separate clones lacking the activated K-*Ras* contained less u-PAR mRNA compared with the parental HCT 116 cells. Thus, HKh-2 and HK2-8 which had the lowest amount of u-PAR protein were characterized as having the least amount of u-PAR mRNA. In contrast, the Hke-3 clone had both intermediate levels of u-PAR protein and mRNA.

### Stimulation of u-PAR expression by PMA and an activated c-Ha-*ras* in HCT 116 cells in which the activated K-*Ras* is disrupted

To rule out the possibility that the method of disrupting the activated K-*Ras* gene in HCT 116 had also physically disrupted the u-PAR gene, we determined if an exogenous stimulus previously shown to be inductive for u-PAR expression (Picone et al, 1989; Lund et al, 1991) elevated the amount of this protein in HKh-2

 *© 1999 Cancer Research Campaign*

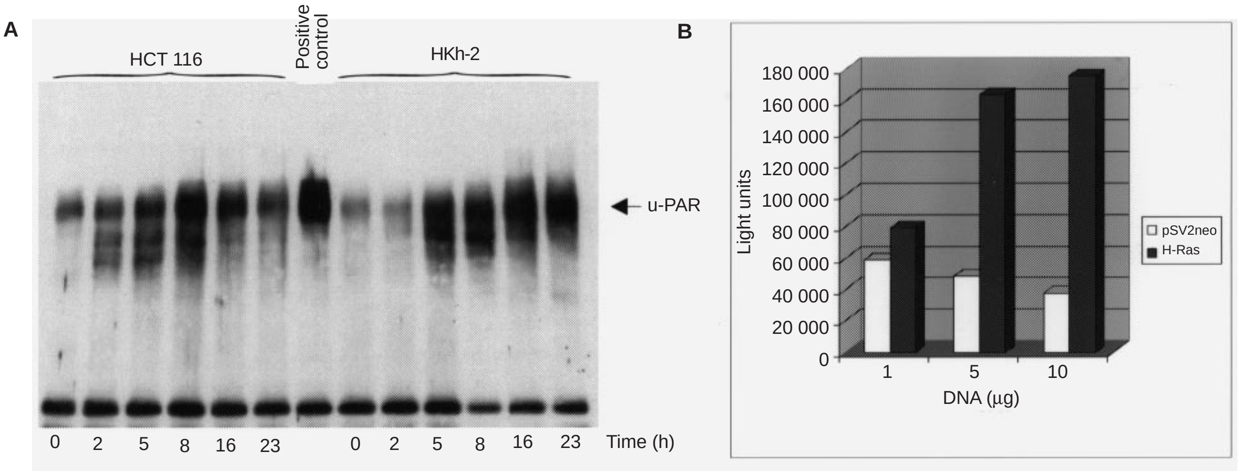

**Figure 2** Phorbol ester induces u-PAR protein synthesis in HCT 116 cells containing a disrupted K-*Ras* oncogene. (**A**) HCT 116 and HKh-2 cells at 80% confluence were treated with PMA (100 nM) for the indicated times after which cellular extracts were generated and analysed for u-PAR protein as described in the legend to Figure 1A. The positive control consists of cellular extract from the RKO cell line which displays 300 000 binding sites per cell (Boyd et al, 1988). (**B**) HKh-2 cells were transfected with a u-PAR luciferase reporter construct and varying amounts of an expression plasmid encoding an activated c-Ha-*ras* gene (H-*Ras*) or, as a control, pSV2neo. After 2 days, the cells were extracted and analysed for luciferase reporter activity cells. Towards this end, HCT 116 and HKh-2 cells were treated with 100 nM PMA for varying periods of time and analysed for u-PAR protein by Western blotting (Figure 2A). Expectedly, in the absence of the phorbol ester, HKh-2 cells contained less u-PAR protein than the parental HCT 116. In contrast, u-PAR protein was substantially induced in both HCT 116 and HKh-2 cells by PMA. Although it appeared that the time frames of induction (maximal inductions 8 and 23 h for HCT 116 and HKh-2 cells respectively) were different, the fact that u-PAR expression was inducible by the phorbol ester indicated that this gene was still subject to control by external stimuli and hence was not physically impaired.

We were also interested in determining whether the re-expression of an activated *Ras* would lead to increased u-PAR expression. Towards this end, HKh-2 cells were co-transfected with a u-PAR promoter-regulated luciferase reporter construct and an activated c-Ha-*ras* (Nicolson et al, 1990) expression plasmid. Increasing amounts of the effector plasmid resulted in a dose-dependent increase in u-PAR promoter activity (Figure 2B). The highest amount of the c-Ha-*ras* expression construct led to over a fivefold induction in u-PAR promoter activity. These results suggested that the signalling machinery connecting the *Ras* protein at the cell surface to nuclear transcription factors regulating u-PAR promoter activity in HKh-2 cells is intact.

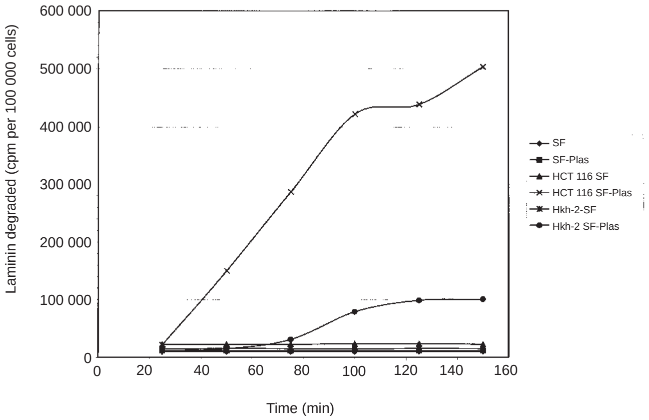

**Figure 3** HCT 116 cells, in which the activated K-*Ras* gene is disrupted, degrades laminin at a slower rate compared with cells containing the activated oncogene. Cells were harvested non-enzymatically and ($10^5$) plated in serum-free medium (SF) onto radioactive laminin-coated dishes. Cells were allowed to attach and cell surface u-PAR saturated by a 30-min incubation with 5 nM exogenous urokinase. After this time, the cells were washed extensively and supplemented with, or without, plasminogen (Plas). The cells were cultured for varying times at 37°C after which aliquots of the culture supernatant were counted for radioactivity. At the end of the experiment, cells were harvested and enumerated. The data are shown as average values of duplicate experiments

### HKh-2 cells degrade laminin at a slower rate than HCT 116 cells

Since one of the functions of the u-PAR is to facilitate plasminogen-dependent proteolysis (Ellis et al, 1991), we determined if laminin degradation by HKh-2 cells was decreased relative to HCT 116 cells. Cells were harvested non-enzymatically and plated on culture dishes coated with radioactive laminin. After cell attachment and saturation of cells surface u-PAR with exogenous urokinase, plasminogen was added and at various times thereafter, aliquots of the culture supernatant were counted for radioactivity. HCT 116 cells rapidly degraded the laminin as measured by the solubilized product. After 100 min, approximately 420 000 cpm of radioactivity was released in duplicate experiments (Figure 3). In contrast, HKh-2 cells solubilized 80% less laminin (about 80 000 cpm) over an identical time frame. These findings are consistent with the view that disruption of the mutated K-Ras in HCT 116 colon cancer cells diminishes u-PAR-directed proteolysis.

### Down-regulation of the u-PAR gene in HCT 116 cells containing a disrupted K-*Ras* oncogene is partly a consequence of reduced transactivation of the promoter through an upstream AP-1 motif

We next determined the mechanism by which u-PAR expression is down-regulated in the K-*Ras* 'knocked out' HCT 116 cells. Since we previously demonstrated the requirement of an upstream AP-1

*© 1999 Cancer Research Campaign* 

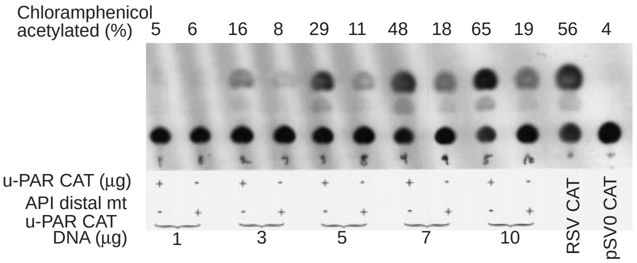

**Figure 4** Requirement of an upstream AP-1 motif for u-PAR expression in HCT 116 cells. HCT 116 cells were transfected with varying amounts of a CAT reporter regulated by either the wild-type (398 base pairs of flanking sequence) u-PAR promoter (u-PAR CAT) or the promoter which had been mutated at the AP-1 motif (AP-1 distal mt u-PAR CAT). Parallel cultures were transfected with RSV CAT and pSV0 CAT as positive and negative controls respectively. After 2 days, the cells were extracted and analysed for CAT reporter activity after normalization for differences in transfection efficiency. Chloramphenicol conversions were determined using a Storm 840 Phorphorimager. The experiment was carried out twice

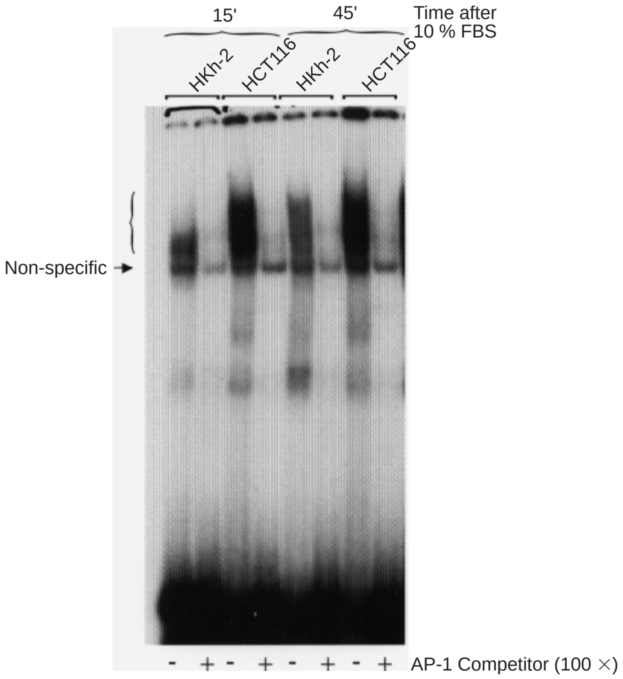

**Figure 5** HCT 116 cells disrupted for the K-*Ras* oncogene demonstrate decreased binding of nuclear factors to the u-PAR promoter AP-1-spanning sequence. Cells were grown to 95% confluence. The culture medium was replenished with fresh FBS-containing medium and, after 15 or 45 min, nuclear extracts generated. Nuclear extracts (15 µg) were incubated with a radioactive oligonucleotide corresponding to the u-PAR promoter sequence spanning nucleotides 199/–170 (thus including the AP-1 motif at –184) in the presence, or absence, of a 100-fold excess of unlabelled oligonucleotide competitor. Binding complexes were resolved by electrophoresis. The data are typical of duplicate experiments.

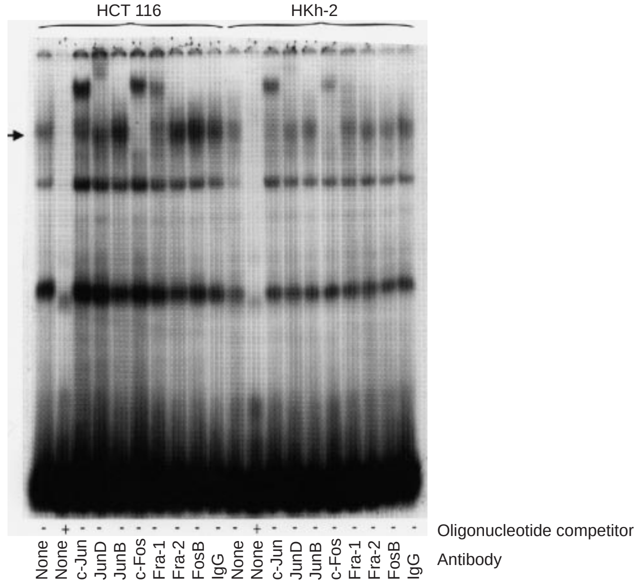

**Figure 6** Decreased binding of c-*Fos*, c-*Jun*, *Jun*D and *Fra*-1 to the u-PAR AP-1 motif in cells disrupted for the K-*Ras* oncogene. Nuclear extract (15 µg) was incubated for 15 min with the oligonucleotide spanning the u-PAR AP-1 motif at –184. Antibodies (1 µg), or as a control IgG (1 µg), were then added and incubated for an additional 10 min. Bound complexes were resolved in a 5% polyacrylamide gel. The data are representative of duplicate experiments motif (184 base pairs upstream of the major transcriptional start site) for the constitutive expression of the gene in another colon cancer cell line (RKO) (Lengyel et al, 1996) we speculated that the high level of u-PAR protein in HCT 116 may similarly be due to transactivation through this motif. To test this possibility, HCT 116 cells were transiently transfected with a CAT reporter driven by either the wild-type or the AP-1-mutated u-PAR promoter. The u-PAR promoter in which the AP-1 motif was mutated, showed a 50–70% reduction compared with the wild-type promoter (Figure 4). Thus, the constitutive activity of the u-PAR promoter in HCT 116 cells is mediated partly through an AP-1 motif at –184.

Since optimal u-PAR expression in HCT 116 required the AP-1 motif at –184, we hypothesized that the amount of transcription factor(s) bound to this motif would be less using nuclear extract from HKh-2 cells in which the mutated K-*Ras* was disrupted. Towards this end, nuclear extracts were generated from both HCT 116 and HKh-2 cells and mobility shifting experiments performed using a u-PAR promoter oligonucleotide spanning the AP-1 motif at –184. Addition of nuclear extract (equal protein) from either HCT 116 or HKh-2 cells resulted in slower moving bands (Figure 5). Most of these were specific (parenthesis) since they were abolished with a 100-fold excess of the non-radioactive oligonucleotide. More importantly, the intensity of the specific bands was substantially greater using nuclear extract from HCT 116 cells compared with that generated with HKh-2 cells. Interestingly, addition of FBS to the HKh-2 cells resulted in some induction in the amount of transcription factor(s) bound (compare 45- and 15-min treatments with 10% FBS). However, this was in the absence of increased u-PAR protein synthesis as evident by Western blotting (Figure 1A). Presumably, this is a consequence of the requirement of multiple transcription factors for induction of u-PAR gene expression. Indeed, we recently reported that u-PAR synthesis is *trans*-activated by an AP-2α-related factor through a separate DNA-binding motif (Allgayer et al, 1999).

To identify the *trans*-acting proteins bound by the oligonucleotide spanning the AP-1 motif (–184) of the u-PAR promoter, supershifting experiments were performed (Figure 6). Addition of antibodies to c-*Jun*, *Jun*D, c-*Fos*, and *Fra*-1 further reduced the mobility of the shifted bands indicating the presence of these AP-1-binding proteins complexed with the u-PAR promoter oligonucleotide. Conversely, antibodies to *Jun*B, *Fra*-2 or *Fos*B failed to supershift the u-PAR promoter oligonucleotide–protein complex. Interestingly, the amount of c-*Jun*, *Jun*D, c-*Fos* and

 © 1999 Cancer Research Campaign

*Fra*-1 bound to the u-PAR promoter oligonucleotide was greater for HCT 116 nuclear extracts when compared with HKh-2 nuclear extracts as judged by the greater intensity of the supershifted bands. Taken together, these data suggest that the lower amount of u-PAR protein in HKh-2 cells containing a disrupted K-*Ras* oncogene is at least partly a consequence of reduced AP-1-dependent transactivation of the promoter. These findings are reminiscent of a recent report demonstrating the accumulation of higher levels (and increased DNA-binding) of the AP-1-binding proteins, c-*Jun* and *Fra*-1 (Mechta et al, 1997) in cells transformed with the K-*Ras* oncogene.

### DISCUSSION

The urokinase receptor has previously been shown to be overexpressed in invasive colon cancer and this overexpression is associated with a shorter survival time for patients afflicted with the cancer (Pyke et al, 1991; Ganesh et al, 1994). However, the initial stimulus for the elevation in u-PAR protein production has yet to be determined. Since, K-*Ras* mutations are common in colon cancer (Bos, 1989; Ahnen et al, 1998) we hypothesized that this activated oncogene contributes to the elevated expression of the urokinase binding site. To answer this question, we compared u-PAR expression in a colon cancer cell line (HCT 116) previously described as having an activated K-*Ras* with HCT 116 cells in which this oncogene was disrupted by homologous recombination. Our data clearly show, for the first time, a reduced expression of the urokinase binding site in the cells in which the activated K-*Ras* gene had been ‘knocked out’. Taken together, these data suggest that u-PAR expression, in at least a sub-population of colon cancer, is regulated by a mutation-activated K-*Ras*. However, it should be emphasized that it is unlikely that elevated u-PAR production in colon cancer is always a consequence of this mutation. Thus, we have found instances in which u-PAR expression in cultured colon cancer is elevated in the absence of a K-*Ras* mutation (Buard et al, 1996; Lengyel et al, 1997).

Interestingly, in an earlier study, Jankun and co-workers (Jankun et al, 1991) had shown an increased amount of urokinase bound to the receptors on fibroblasts transformed with the K-*Ras* oncogene. The authors in that publication concluded that the K-*Ras* oncogene was increasing the expression of the serine protease thereby accounting for the increased occupation of the binding sites. At the same time, it is equally possible that the transformed fibroblasts were manifesting elevated u-PAR expression thereby capturing more of the protease. However, since u-PAR expression was not measured directly, it is not clear whether the K-*Ras* oncogene increased production of the u-PAR in the fibroblasts.

Although, the u-PAR expression was reduced by the disruption of the activated K-*Ras* gene, by no means was expression eliminated. These findings would indicate that other signalling mechanisms also contribute to u-PAR expression in the HCT 116 colon cancer cells. For example, it is possible that growth factor signalling through the normal K-*Ras* allele (which is intact) maintains u-PAR expression. However, this is a less likely possibility since we found that u-PAR expression was unchanged by growing the cells in the absence of serum. Alternatively, it may very well be that signalling events downstream (or in parallel to) (Herrera-Velit et al, 1997) of K-*Ras* also contribute to the elevated expression of the urokinase binding site in HCT 116 colon cancer cells.

Considering the evidence implicating the activated K-*Ras* in regulating u-PAR expression, what is the molecular mechanism by which this is accomplished? For u-PAR expression, the current and a previous study (Lengyel et al, 1996) have implicated an upstream AP-1 motif required for the constitutive expression of the gene in invasive cultured colon cancer. There has been intense effort to elucidate the downstream molecules that transmit the signal from *Ras* to the transcription factors regulating AP-1-dependent gene expression. For example, it is well recognized that *Ras* can signal through the classical c-*Raf*-1-MEK1, extracellular signal-regulated kinase (ERK) cascade (Minden, et al 1994) as well as through the parallel *Rac*-1, mitogen-activated protein kinase kinase (MEKK), c-*Jun* amino-terminal kinase (JNK) pathway (Minden et al, 1994; Russell et al, 1995) or a separate PI-3 kinase-dependent pathway (Rodriguez-Viciana et al, 1994; Winkler et al, 1997). Indeed, it is possible that the K-*Ras*-regulated u-PAR expression in HCT 116 cells is ERK-dependent. Thus, we previously reported that down-regulation of u-PAR expression in another colon cancer cell line (RKO) could be achieved by the expression of a dominant negative expression construct to ERK1 or by treatment of cells with an inhibitor of ERK1 activation (PD 098059) (Lengyel et al, 1997). Consistent with this notion is the finding by us of increased amount of c-*Fos* bound to the u-PAR promoter AP-1 motif. It is well known that c-*Fos* expression is increased by the ERKs via stimulation of ternary complex formation on the c-*Fos* promoter (Gille et al, 1992). On the other hand, recent studies have shown that the activated K-*Ras* in HCT 116 cells does not result in a constitutive activation of MEK1 and ERK (Ohmori et al, 1997). Thus, it may very well be that other signalling modules in HCT 116 cells are required for regulating u-PAR expression in response to the K-*Ras* oncogene. For example, the involvement of a JNK1-dependent signalling module in the regulation of u-PAR expression by phorbol ester was demonstrated by our group (Gum et al, 1998). This MAPK increased AP-1-dependent gene transcription largely by increasing the transcriptional activity of c-*Jun* subsequent to its phosphorylation on serine residues (Hibi et al, 1993; Adler et al, 1994; Minden et al, 1994). Again, however, previous studies from Ohmori et al (1997) would indicate otherwise, Thus, these workers found that JNK was not activated in HCT 116 cells when compared with the clones Hke-3 and HKh-2 in which K-*Ras* was deleted by homologous recombination. We can only speculate that the reduced u-PAR expression in the K-*Ras*-knocked out cells is due to another signalling pathway. One potential cascade could be p38, which regulates c-*Fos* expression via preventing the activation of the Sap-1A transcription factor (Janknecht et al, 1997). Alternatively, it may be that the increased c-*myc* levels (Ohmori et al, 1997) in HCT 116 cells are related, in some way, to the elevated u-PAR levels evident in this cell line when compared with clones in which the K-*Ras* gene was disrupted.

In conclusion we have, for the first time, provided evidence for a role of an activated K-*Ras* as a regulator of the overexpression of the urokinase binding site and consequently plasminogen-dependent extracellular matrix degradation in at least these cultured colon cancer cell lines.

### ACKNOWLEDGEMENTS

We express our appreciation to Dr Yao Wang, The University of Sydney, Westmead, Australia for supplying the u-PAR CAT construct. We thank Dr Andrew Mazar (Angstrom

© 1999 Cancer Research Campaign

Pharmaceuticals) for the generous gift of the u-PAR antibody. We are grateful to Hector Avila and Parham Khanbolooki for excellent technical assistance. This work was supported by NIH grants ROI DE10845, ROI CA58311, P50 DE11906 to DB, and a fellowship to HA (Dr Mildred Scheel Cancer Foundation, Deutsche Krebshilfe, Bonn, Germany).

### REFERENCES

Adler V, Unlap T and Kraft AS (1994) A peptide encoding the c-Jun δ domain inhibits the activity of a c-Jun amino-terminal protein kinase. *J Biol Chem* **269**: 11186–11191

Ahnen DJ, Feigl P, Quan G, Fenoglio-Preiser C, Lovato LC, Bunn PA, Stemmerman G, Wells JD, MacDonald JS and Meyskens FL (1998) Ki-ras mutation and p53 overexpression predict the clinical behavior of colorectal cancer: a southwest oncology group study. *Cancer Res* **58**: 1149–1158

Allgayer H, Heiss MM, Riesenberg R, Grutzner KU, Tarabichi A, Babic R and Schildberg FW (1997) Urokinase plasminogen activator receptor (uPA-R): one potential characteristic of metastatic phenotypes in minimal residual tumor disease. *Cancer Res* **57**: 1394–1399

Allgayer H, Wang H, Wang Y, Heiss MM, Bauer R, Nyormoi O and Boyd D (1999) Transactivation of the urokinase-type plasminogen activator receptor gene through a novel promoter motif bound with an activator protein-2α-related factor. *J Biol Chem* **274**: 4702–4714

Behrendt N, Ploug M, Patthy L, Houen G, Blasi F and Dano K (1991) The ligand-binding domain of the cell surface receptor for urokinase-type plasminogen activator. *J Biol Chem* **266**: 7842–7847

Bohuslav J, Horejsi V, Hansmann C, Stockl J, Weidle UH, Majdic O, Bartke I, Knapp W and Stockinger H (1995) Urokinase plasminogen activator receptor, B2-integrins, and Src-kinases within a single receptor complex of human monocytes. *J Exp Med* **181**: 1381–1390

Bos JL (1989) *ras* oncogenes in human cancer: a review. *Cancer Res* **49**: 4682–4689

Boyd D, Florent G, Kim P and Brattain MG (1988) Determination of the levels of urokinase and its receptor in human colon carcinoma cell lines. *Cancer Res* **48**: 3112–3116

Buard A, Zipfel PA, Frey RS and Mulder KM (1996) Maintenance of growth factor signalling through Ras in human colon carcinoma cells containing K-*ras* mutations. *Int J Cancer* **67**: 539–546

Burnette W (1981) “Western Blotting” Electrophoretic transfer of proteins from sodium dodecyl sulfate polyacrylamide gels to unmodified nitrocellulose and radiographic detection with antibody and radioiodinated protein A. *Analyt Biochem* **112**: 195–203

Casey JR, Petranka JG, Kottra J, Fleenor DE and Rosse WF (1994) The structure of the urokinase-type plasminogen activator receptor gene. *Blood* **84**: 1151–1156

Conese M, Olson D and Blasi F (1994) Protease nexin-1-urokinase complexes are internalized and degraded through a mechanism that requires both urokinase receptor and $\alpha_2$-macroglobulin receptor. *J Biol Chem* **269**: 17886–17892

Cubellis M, Wun T and Blasi F (1990) Receptor-mediated internalization and degradation of urokinase is caused by its specific inhibitor PAI-1. *Eur Mol Biol Organ* **9**: 1079–1085

Ellis V, Behrendt N and Dano K (1991) Plasminogen activation by receptor-bound urokinase. *J Biol Chem* **266**: 12,752–12,758

Ganesh S, Sier CFM, Heerding MM, Griffioen G, Lamers CBH and Verspaget HW (1994) Urokinase receptor and colorectal cancer survival. *Lancet* **344**: 401–402

Gille H, Sharrocks AD and Shaw PE (1992) Phosphorylation of transcription factor $p62^{tcf}$ by MAP kinase stimulates ternary complex formation at c-*fos* promoter. *Nature* **358**: 414–417

Gum R, Juarez J, Allgayer H, Mazar A, Wang Y and Boyd D (1998) Stimulation of urokinase-type plasminogen activator receptor expression by PMA requires JNK1-dependent and -independent signaling modules. *Oncogene* **17**: 213–226

Herrera-Velit P, Knutson KL and Reiner NE (1997) Phosphatidylinositol 3-kinase-dependent activation of protein kinase C-zeta in bacterial lipopolylsaccharide-treated human monocytes. *J Biol Chem* **272**: 16445–16452

Hibi M, Lin A, Minden A and Karin M (1993) Identification of an oncoprotein- and uv-responsive protein kinase that binds and potentiates the c-Jun activation domain. *Genes Dev* **7**: 2135–2148

Higazi A, Cohen R, Henkin J, Kniss D, Schwartz BS and Cines D (1995) Enhancement of the enzymatic activity of single-chain urokinase plasminogen activator by soluble urokinase receptor. *J Biol Chem* **270**: 17375–17380

Hollas W, Blasi F and Boyd D (1991) Role of the urokinase receptor in facilitating extracellular matrix invasion by cultured colon cancer. *Cancer Res* **51**: 3690–3695

Janknecht OR and Hunter T (1997) Activation of the Sap-1a transcription factor by the c-Jun N-terminal kinase (JNK) mitogen-activated protein kinase. *J Biol Chem* **272**: 4219–4224

Jankun J, Maher V and McCormick. J (1991) Malignant transformation of human fibroblasts correlates with increased activity of receptor-bound plasminogen activator. *Cancer Res* **51**: 1221–1226

Kariko K, Kuo A, Boyd D, Okada S, Cines D and Barnathan E (1993) Overexpression of urokinase receptor increases matrix invasion without altering cell migration in a human osteosarcoma cell line. *Cancer Res* **53**: 3109–3117

Kook YH, Adamski J, Zelent A and Ossowski L (1994) The effect of antisense inhibition of urokinase receptor in human squamous cell carcinoma on malignancy. *Eur Mol Biol Organ* **13**: 3983–3991

Lengyel E, Wang H, Gum R, Simon C, Wang Y and Boyd D (1997) Elevated urokinase-type plasminogen activator receptor expression in a colon cancer cell line is due to a constitutively activated extracellular signal-regulated kinase-1-dependent signaling cascade. *Oncogene* **14**: 2563–2573

Lengyel E, Wang H, Stepp E, Juarez J, Doe W, Pfarr CM and Boyd D (1996) Requirement of an upstream AP-1 motif for the constitutive and phorbol ester-inducible expression of the urokinase-type plasminogen activator receptor gene. *J Biol Chem* **271**: 23176–23184

Lund L, Ronne E, Roldan A, Behrendt N, Romer J, Blasi F and Dano K (1991) Urokinase receptor mRNA level and gene transcription are strongly and rapidly increased by phorbol myristate acetate in human monocyte-like U937 cells. *J Biol Chem* **266**: 5177–5181

Lund LR, Ellis V, Ronne E, Pyke C and Dano K (1995) Transcriptional and post-transcriptional regulation of the receptor for urokinase-type plasminogen activator by cytokines and tumor promoters in the human lung carcinoma cell line A549. *Biochem J* **310**: 345–352

Lund LR, Ellis V, Ronne E, Pyke C and Dano K (1995) Transcriptional and post-transcriptional regulation of the receptor for urokinase-type plasminogen activator by cytokines and tumor promoters in the human lung carcinoma cell line A549. *Biochem J* **310**: 345–352

Mechta F, Lallemand D, Pfarr CM and Yaniv M (1997) Transformation by ras modifies AP1 composition and activity. *Oncogene* **14**: 837–847

Minden A, Lin A, McMahon M, Lange-Carter C, Derijard B, Davis RJ, Johnson GL and Karin M (1994*a*) Differential activation of ERK and JNK mitogen-activated protein kinases by raf-1 and MEKK. *Science* **266**: 1719–1722

Minden A, Lin A, Smeal T, Derijard B, Cobb M, Davis R and Karin M (1994*b*) c-Jun N-terminal phosphorylation correlates with activation of the JNK subgroup but not the ERK subgroup of mitogen-activated protein kinases. *Mol Cell Biol* **14**: 6683–6688

Moller L, Pollanen J, Ronne E, Pedersen N and Blasi F (1993) N-linked glycosylation of the ligand-binding domain of the human urokinase receptor contributes to the affinity for its ligand. *J Biol Chem* **268**: 11152–11159

Nead MA and McCance DJ (1995) Poly-L-ornithine-mediated transfection of human keratinocytes. *J Invest Dermatol* **105**: 668–671

Nicolson GL, Gallick GE, Dulski K, Spohn WH, Lembo TM and Tainsky MA (1990) Lack of correlation between intercellular junctional communication, p21 $ras^{EJ}$ expression, and spontaneous metastatic properties of rat mammary cells after transfection with c-Ha-ras or *neo* genes. *Oncogene* **5**: 747–753

Nielsen L, Hansen J, Skriver L, Wilson E, Kaltoft K, Zenthen J and Dano K (1982) Purification of zymogen to plasminogen activator from human glioblastoma cells by affinity chromatography with monoclonal antibody. *Biochemistry* **21**: 6410–6415

Nykjaer A, Conese M, Cremona O, Gliemann J and Blasi F (1997) Recycling of the urokinase receptor upon internalization of the uPA:serpin complexes. *Eur Mol Biol Organ* **16**: 2610–2620

Ohmori M, Shirasawa S, Furuse M, Okumura K and Sasazuki T (1997) Activated Ki-*ras* enhances sensitivity of ceramide-induced apoptosis without c-Jun NH2-terminal kinase/stress-activated protein kinase or extracellular signal-regulated kinase activation in human colon cancer cells. *Cancer Res* **57**: 4714–4717

Picone R, Kajtaniak E, Nielsen L, Behrendt N, Mastronicola M, Cubellis MV, Stoppelli MP, Pedersen S, Dano K and Blasi F (1989) Regulation of urokinase receptors in monocyte like cells U937 by phorbol ester phorbol myristate acetate. *J Cell Biol* **108**: 693–702

Pyke C, Kristensen P, Ralfkiaer E, Grondahl-Hansen J, Eirksen J, Blasi F and Dano K (1991) Urokinase-type plasminogen activator is expressed in stromal cells and its receptor in cancer cells at invasive foci in human colon adenocarcinomas. *Am J Pathol* **138**: 1059–1067

Pyke C, Eriksen J, Solberg H, Schnack B, Nielsen S, Kristensen P, Lund LR and Dano K (1993) An alternatively spliced variant of mRNA for the human receptor for urokinase plasminogen activator. *FEBS* **326**: 69–74

 © 1999 Cancer Research Campaign

Quattrone A, Fibbi G, Anichini E, Pucci M, Zamperini A, Capaccioli S and Del Rosso M (1995) Reversion of the invasive phenotype of transformed human fibroblasts by anti-messenger oligonucleotide inhibition of urokinase receptor gene expression. *Cancer Res* **55**: 90–95

Riittinen L, Limongi P, Crippa MP, Conese M, Hernandez-Marrero L, Fazioli F and Blasi F (1996) Removal of domain D2 or D3 of the human urokinase receptor does not affect ligand affinity. *FEBS Lett* **381**: 1–6

Robbins KC, Summaria L, Hsieh B and Shah R (1967) The peptide chains of human plasmin. *J Biol Chem* **242**: 2333–2342

Rodriguez-Viciana P, Warne PH, Dhand R, Vanhaesebroeck B, Gout I, Fry MJ, Waterfield MD and Downward J (1994) Phosphatidylinositol-3-OH kinase as a direct target of ras. *Nature* **370**: 527–532

Roldan A, Cubellis M, Masucci M, Behrendt N, Lund L, Dano K, Appella E and Blasi F (1990) Cloning and expression of the receptor for urokinase plasminogen activator, a central molecule in cell surface, plasmin dependent proteolysis. *Eur Mol Biol Organ* **9**: 467–474

Russell M, Lange-Carter CA and Johnson GL (1995) Direct interaction between ras and the kinase domain of mitogen-activated protein kinase kinase (MEKK1). *J Biol Chem* **270**: 11757–11760

Schlechte W, Murano G and Boyd DD (1989) Examination of the role of the urokinase receptor in human colon cancer mediated laminin degradation. *Cancer Res* **49**: 6064–6069

Shetty S, Kumar A and Idell S (1997) Posttranscriptional regulation of urokinase receptor mRNA: identification of a novel urokinase receptor mRNA binding protein in human mesothelioma cells. *Mol Cell Biol* **17**: 1075–1083

Shirasawa S, Furuse M, Yokoyama N and Sasazuki T (1993) Altered growth of human colon cancer cell lines disrupted at activated. Ki-*ras Science* **260**: 85–88

Stoppelli MP, Tacchetti C, Cubellis M, Corti A, Hearing V, Cassani G, Appella E and Blasi F (1986) Autocrine saturation of pro-urokinase receptors on human A431 cells. *Cell* **45**: 675–684

Vagnarelli P, Raimondi E, Mazzieri R, De Carli L and Migantti P (1992) Assignment of the human urokinase receptor gene (PLAU) to 19q13. *Cytogenet Cell Genet* **60**: 197–199

Vassalli J, Baccino D and Belin D (1985) A cellular binding site for the Mr 55,000 form of the human plasminogen activator, urokinase. *J Cell Biol* **100**: 86–92

Wang Y, Dang J, Johnson LK, Selhamer JJ and Doe WF (1995) Structure of the human urokinase receptor gene and its similarity to CD59 and the Ly-6 family. *Eur J Biochem* **227**: 116–122

Wei Y, Lukashev M, Simon DI, Bodary SC, Rosenberg S, Doyle MV and Chapman HA (1996) Regulation of integrin function by the urokinase receptor. *Science* **273**: 1551–1555

Wilhelm O, Weilde U, Rettenberger S, Schmitt M and Graeff H (1994) Recombinant soluble urokinase receptor as a scavenger for urokinase-type plasminogen activator (uPA). Inhibition of proliferation and invasion of human ovarian cancer cells. *FEBS Lett* **337**: 131–134

Winkler DG, Johnson JC, Cooper JA and Vojtek AB (1997) Identification and characterization of mutations in Ha-Ras that selectively decrease binding to cRaf-1. *J Biol Chem* **272**: 24402–24409

Yebra M, Parry GCN, Stromblad S, Mackman N, Rosenberg S, Mueller BM and Cheresh DA (1996) Requirement of receptor-bound urokinase-type plasminogen activator for integrin $\alpha v\beta$ 5-directed cell migration. *J Biol Chem* **271**: 29393–29399

*© 1999 Cancer Research Campaign*
